# Inferior and medial temporal tau and cortical amyloid are associated with daily functional impairment in Alzheimer’s disease

**DOI:** 10.1186/s13195-019-0471-6

**Published:** 2019-01-31

**Authors:** Omar A. Halawa, Jennifer R. Gatchel, Rebecca E. Amariglio, Dorene M. Rentz, Reisa A. Sperling, Keith A. Johnson, Gad A. Marshall, Alzheimer’s Disease Neuroimaging Initiative

**Affiliations:** 1 Harvard Medical School, Boston, MA 02115 USA; 2 Department of Psychiatry, Massachusetts General Hospital, Boston, MA 02114 USA; 3 Department of Neurology, Massachusetts General Hospital, Boston, MA 02114 USA; 4 Department of Radiology, Massachusetts General Hospital, Boston, MA 02114 USA; 5 Division of Geriatric Psychiatry, McLean Hospital, Belmont, MA 02478 USA; 6 Center for Alzheimer Research and Treatment, Brigham and Women’s Hospital, 60 Fenwood Road, 9016P, Boston, MA 02115 USA; 7 Department of Neurology, Brigham and Women’s Hospital, Boston, MA 02115 USA; 8 Department of Psychiatry, Brigham and Women’s Hospital, Boston, MA 02115 USA

**Keywords:** Alzheimer’s disease, Instrumental activities of daily living, Tau, Amyloid, Positron emission tomography, Mild cognitive impairment, Inferior temporal cortex, Entorhinal cortex

## Abstract

**Background:**

A decline in instrumental activities of daily living (IADL) correlates with the progression from mild cognitive impairment (MCI) to Alzheimer’s disease (AD) dementia and has been associated with frontal and parietal hypometabolism, lower cerebrospinal fluid amyloid *β*_1–42_, and inferior temporal cortical thinning. Identifying the underlying biomarkers of functional decline will allow for the early identification of individuals at risk of disease progression.

**Objective:**

To investigate the association between IADL impairment and in vivo regional cerebral tau and cortical amyloid deposition across clinically normal (CN) elderly, MCI, and AD dementia.

**Methods:**

Fifty-one CN elderly, 30 MCI, and 9 AD dementia participants of the Alzheimer’s Disease Neuroimaging Initiative (ADNI) underwent assessment of regional tau deposition with flortaucipir (FTP) positron emission tomography (PET). An aggregate of cortical amyloid burden was assessed by florbetapir PET. IADL were assessed using the Functional Activities Questionnaire (FAQ). Tau regions with unadjusted correlations of *p* ≤ 0.006 (Bonferroni correction) with FAQ were used to evaluate the cross-sectional association between FAQ (dependent variable) and regional cerebral tau deposition, amyloid burden, and tau-amyloid interaction in separate general linear regression models with backward elimination. Covariates included age, American National Adult Reading Test (AMNART) intelligence quotient (IQ), and Rey Auditory Verbal Learning Test (RAVLT) total learning.

**Results:**

Unadjusted correlations between FAQ and tau in the entorhinal cortex (EC) and inferior temporal cortex (IT) survived Bonferroni correction. FAQ was associated with the tau-amyloid interaction, such that in participants with greater amyloid burden, greater IADL impairment was associated with greater regional tau (EC tau × amyloid: partial *r* (pr) = 0.47, *p* < 0.001; IT tau × amyloid: pr = 0.54, *p* < 0.001). Significant associations were found when these regression models were repeated in symptomatic participants alone but not among CN participants.

**Conclusions:**

Greater medial and inferior temporal tau and cortical amyloid burden were associated with greater IADL impairment in AD. Further elucidation of the biomarkers underlying the functional decline will allow for the early identification of individual at risk of disease progression.

## Background

Alzheimer’s disease (AD) affected 5.2 million Americans in 2016, with the number of those diagnosed estimated to reach 13.8 million by 2050 [1]. Progression of the disease from the clinically normal (CN) stage to mild cognitive impairment (MCI), followed by AD dementia, is marked by worsening memory and cognition, as well as impairment in daily functioning [2, 3]. Efforts have been made to identify imaging and neuropathologic and clinical correlates of the stages of AD dementia, with the purpose of providing early interventions to slow disease progression. Functional impairment is one correlate of disease progression that can be measured clinically and that can take a significant toll on a patient’s quality of life, increasing the burden on caregivers [4]. While patients in the moderate to late stage of AD dementia will have already lost the ability to carry out basic tasks such as grooming and bathing, known as basic activities of daily living (ADL), impairment in more complex functional activities such as managing finances and shopping for groceries, known as instrumental ADL (IADL), occurs earlier, marking the progression from MCI to AD dementia [2, 5, 6]. Further investigation of IADL decline across the AD spectrum, including CN subjects, may allow for the detection of disease progression earlier.

Several neuropathologic and neuroimaging correlates of IADL impairment have been identified. Frontal and parietal hypometabolism, lower cerebrospinal fluid amyloid *β*_1–42_, inferior temporal cortical thinning, and bilateral prefrontal-striate-anterior cingulate hypoperfusion have all been associated with greater IADL impairment across the stages of AD [7–13]. The finding that there is a relationship between IADL impairment and pathologic markers even among CN participants points to the potential role of IADL in identifying individuals at risk of decline early in the disease progression. Importantly, the association between IADL and pathologic markers has been shown to be independent of cognitive function (measured by the Rey Auditory Verbal Learning Task (RAVLT) and the Mini-Mental State Examination (MMSE)) and premorbid intelligence (measured by the American National Adult Reading Test (AMNART)) [7, 9].

Tau accumulation has been identified as a marker of neurodegeneration and cognitive impairment in several neurodegenerative diseases [14]. With the emergence of flortaucipir (FTP) (a.k.a. AV-1451) positron emission tomography (PET) measurements tracking tau uptake and their incorporation in the third phase of the Alzheimer’s Disease Neuroimaging Initiative (ADNI3), several studies have explored the link between tau accumulation in the context of amyloid deposition and other neuroimaging and neuropathologic correlates [15–20]. Studying the relationship between regional tau deposition and IADL impairment will serve to explore the utility of IADL impairment in tracking and predicting disease progression. The objective of this study, therefore, was to investigate the associations among IADL and in vivo regional cerebral tau and cortical amyloid deposition across CN elderly, MCI, and AD dementia. Given the previously reported involvement of the frontal, parietal, and inferior temporal regions with IADL impairment visualized with other imaging modalities and at post-mortem, we hypothesized that greater tau burden in those regions together with cortical amyloid burden would correlate with greater IADL impairment [7–9, 12, 21]. We further wanted to demonstrate that the association between IADL impairment and tau and amyloid burden is independent of cognitive function and a proxy of cognitive reserve (premorbid intelligence).

## Methods

### Participants

Data used in the preparation of this article were obtained from the ADNI database (adni.loni.usc.edu). ADNI was launched in 2003 as a public-private partnership, led by principal investigator Michael W. Weiner, MD. The primary goal of ADNI has been to test whether serial MRI, PET, other biological markers, and clinical and neuropsychological assessment can be combined to measure the progression of MCI and early AD. Data used in the current study were collected on 51 CN elderly, 30 amnestic MCI, and 9 AD dementia participants taking part in the third phase of ADNI. This sample represents the ADNI participants who have undergone tau PET imaging, a recent addition to the ADNI protocol. Participants were 62–94 years old, were 56.7% female, and scored between 14 and 30 on the Mini-Mental State Examination (MMSE) [22]. At each site, the principal investigator or a co-investigator experienced in assessing patients with dementia determined the participant’s diagnosis based on a review of the available tests of cognition, IADL, and neuropsychiatric symptoms. Data analyzed for this study were obtained from the initial clinical assessments and imaging data of the ADNI3 protocol. ADNI3, the third phase of ADNI, began in 2016 with changes and additions to the biomarkers and clinical assessments used in ADNI2. Participants in this sample were all previously enrolled in ADNI2 and underwent FTP (a.k.a. AV-1451) tau PET and florbetapir (a.k.a. AV-45) amyloid PET imaging, as well as additional clinical assessments as part of ADNI3. Rollover participants from ADNI2 must have been enrolled in ADNI previously and willing to continue the participation. Inclusion and exclusion criteria used for enrollment in ADNI2 are outlined in the ADNI Procedures Manual (http://adni.loni.usc.edu/wp-content/uploads/2008/07/adni2-procedures-manual.pdf).

### Clinical assessments

IADL were assessed using the study partner-reported 10-item Functional Activities Questionnaire (FAQ) [4]. Ten IADL items were scored (0–3) based on the degree of dependence, yielding a total score of 0–30 with a higher score indicating greater impairment. The IADL items ranged from basic tasks such as heating water to more complex tasks such as assembling tax records. Premorbid verbal intelligence was measured using the American National Adult Reading Test (AMNART) intelligence quotient (IQ) [23]. AMNART IQ scores range from 74 to 132, with a higher score indicating higher premorbid intelligence. The Rey Auditory Verbal Learning Test (RAVLT) total learning score was used as a measure of episodic memory performance [24]. RAVLT scores range from 0 to 75, with lower scores indicating worse performance. The Mini-Mental State Examination (MMSE) was used as a measure of global cognitive function and was used to replace the RAVLT as a covariate in the secondary analyses, in order to correct for differences in cognitive domains not assessed by the RAVLT and thus not adjusted for in our primary analyses, as well as serve as a proxy of disease severity [22]. MMSE scores range from 0 to 30, with lower scores indicating worse cognitive function.

### Amyloid and tau region of interest generation and selection

Florbetapir and FTP PET summary data were obtained from the ADNI Laboratory of Neuroimaging (LONI) database, as previously described [25]. In brief, a native-space MRI scan was used for each participant that is segmented and parcellated with Freesurfer (version 5.3.0) to define the amyloid cortical gray matter regions of interest (frontal, anterior/posterior cingulate, lateral parietal, lateral temporal) that make up a summary cortical regions of interest (ROI) (UC Berkeley—AV45 Analysis Methods, ADNI). The reference region used was the whole cerebellum. An aggregate of cortical amyloid burden was calculated by finding the mean composition score of these four cortical ROIs. Amyloid status was determined using a cutoff of florbetapir SUVR of 1.40. This cutoff was determined based on the distribution of florbetapir uptake across our entire sample. All AD participants had a florbetapir SUVR uptake greater or equal to 1.40. Across CN and MCI participants, there was a bimodal distribution of SUVR uptake, with the majority of participants falling below or above 1.40.

Each FTP scan was co-registered to the corresponding MRI scan, segmented and parcellated with Freesurfer version 5.3.0, to calculate the mean flortaucipir uptake within each Freesurfer-defined region (UC Berkeley—AV1451 Analysis Methods, ADNI). The reference region used was the cerebellar cortex. Eight cortical and subcortical tau ROIs were selected based on their correlation to IADL in previous studies [7–9, 12, 21]: bilateral entorhinal cortex, inferior temporal cortex, rostral anterior cingulate cortex, posterior cingulate cortex, supramarginal gyrus, orbitofrontal cortex, precuneus, and dorsolateral prefrontal cortex. In primary analyses, a tau-amyloid interaction variable was created by multiplying tau deposition in each region of interest with the aggregate cortical amyloid score.

### Statistical analyses

SPSS version 24.0 (IBM Corp., Armonk, NY) was used in all analyses. Unadjusted Spearman correlations between FAQ and tau regions were performed. Those with *p* < 0.006 (Bonferroni correction) were then assessed in our primary analyses. Separate general linear regression models with backward elimination (*p <* 0.1 retention requirement; *p* < 0.05 considered significant) were employed to evaluate the cross-sectional association between FAQ (dependent variable) and regional cerebral tau deposition, amyloid burden, and tau-amyloid interaction. Covariates included age, AMNART IQ, RAVLT total learning, and the MMSE. Partial regression correlations (pr) and significance test results (*p* values) were reported for each predictor retained.

## Results

Participant demographic, clinical and neuropsychological testing characteristics, in addition to amyloid-positive status and FTP and florbetapir uptake means are listed in Table 1. Unadjusted Spearman correlations between FAQ and tau deposition in the entorhinal cortex (EC) and inferior temporal cortex (IT) survived Bonferroni correction (EC: *r*_*s*_ = 0.43, *p <* 0.001; IT: *r*_*s*_ = 0.31, *p* = 0.003).

**Table 1:** Participant demographic and neuropsychological testing characteristics

Diagnosis	All	CN	MCI/AD
*n*	90	51	39
Age (years)	76.3 ± 6.9	74.5 ± 6.1	78.6 ± 7.3
Gender (% female)	56.7	49.0	66.7
Education (years)	16.3 ± 2.6	16.2 ± 2.3	16.4 ± 3.0
AMNART IQ	118.5 ± 9.8	120.0 ± 7.9	116.0 ± 11.7
MMSE	27.8 ± 3.3	29.0 ± 1.2	26.0 ± 4.4
RAVLT total learning	41.4 ± 13.9	48.0 ± 10.7	33.0 ± 13.4
FAQ	3.2 ± 6.4	0.4 ± 1.6	7.0 ± 8.2
Amyloid status (% amyloid-positive)	34.6	22.9	54.5*
Entorhinal Cortex FTP SUVR	1.17 ± 0.21	1.13 ± 0.17	1.24 ± 0.23
Inferior temporal cortex FTP SUVR	1.26 ± 0.26	1.20 ± 0.17	1.33 ± 0.33
Mean cortical florbetapir SUVR	1.37 ± 0.27	1.31 ± 0.22	1.4 ± 0.3

*MCI 40% amyloid-positive, AD dementia 100% amyloid-positive

### Primary analyses

**Fig. 1:**
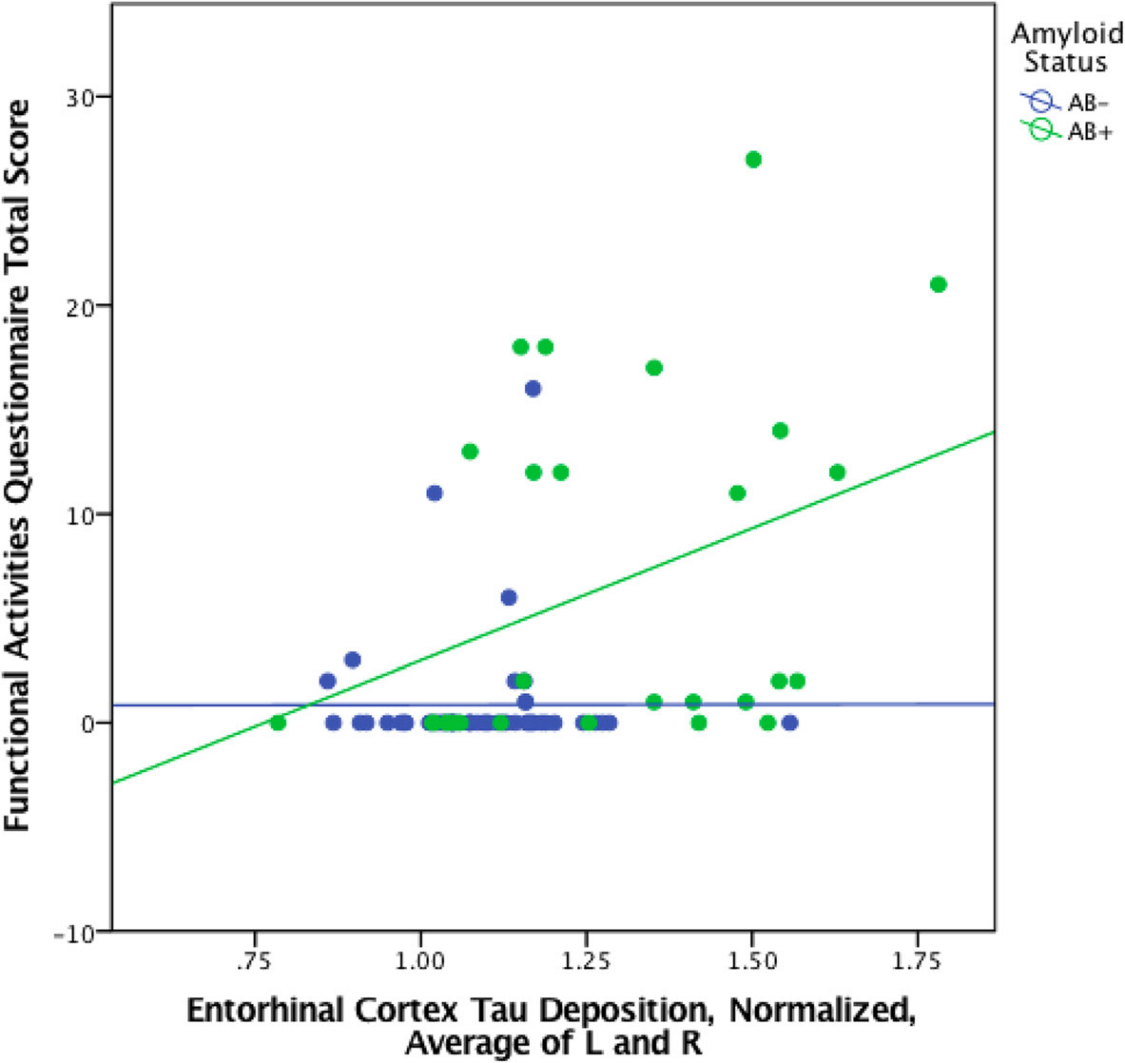
Scatter plot of entorhinal cortex FTP SUVR versus FAQ in CN, MCI, and AD dementia participants, stratified by amyloid status (Aβ+, florbetapir SUVR ≥ 1.40; Aβ−, florbetapir SUVR < 1.40)

**Fig. 2:**
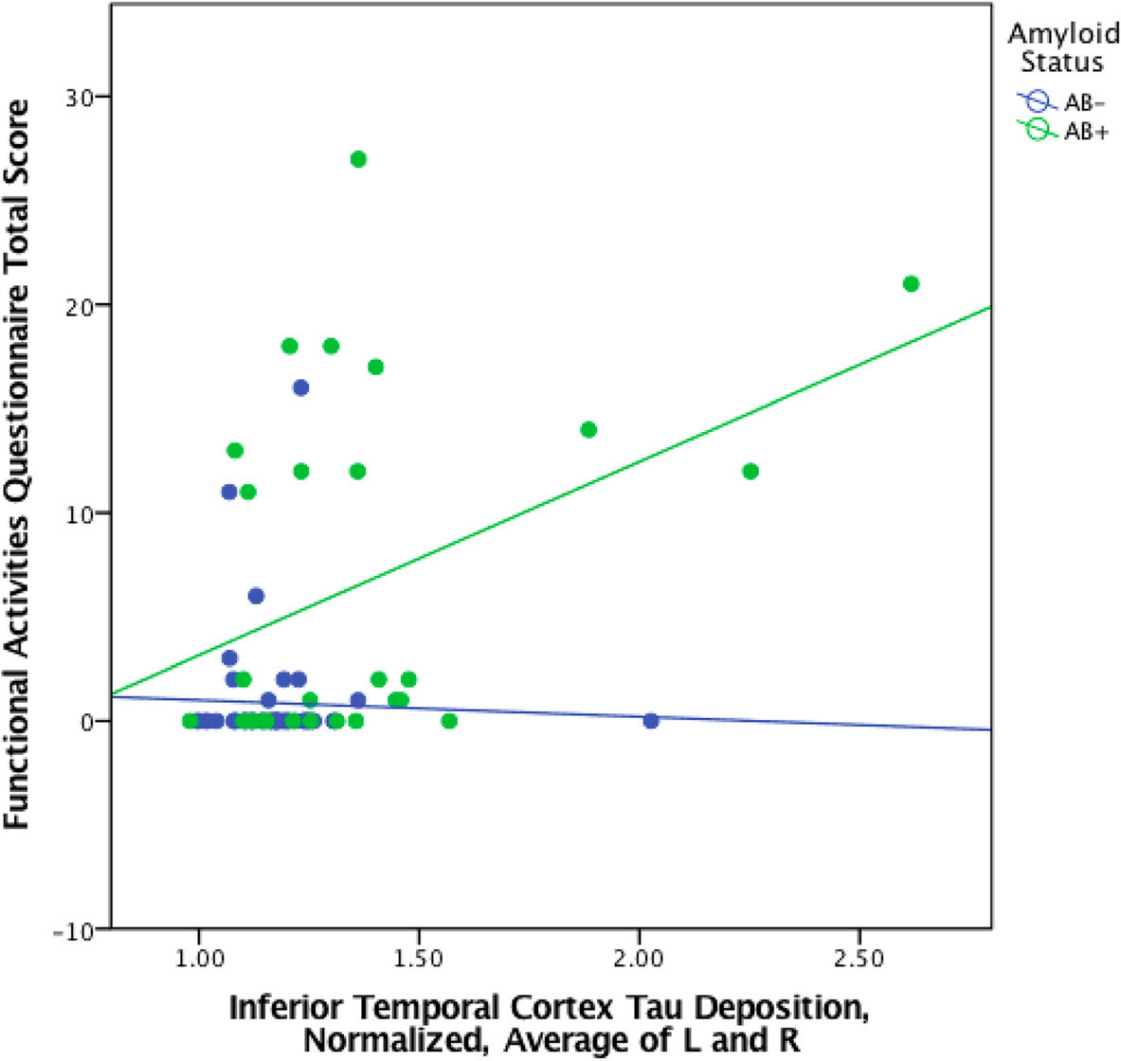
Scatter plot of inferior temporal cortex FTP SUVR versus FAQ in CN, MCI, and AD dementia participants, stratified by amyloid status (Aβ+, florbetapir SUVR ≥ 1.40; Aβ−, florbetapir SUVR < 1.40)

Subsequent separate regression models for EC or IT tau yielded significant associations between FAQ and tau-amyloid interaction, such that in individuals with greater amyloid burden, there was a significant association between greater IADL impairment (higher FAQ score) and greater regional tau burden (EC tau × amyloid: partial *r* (pr) = 0.47, *p* < 0.001; IT tau × amyloid: pr = 0.54, *p* < 0.001) (see Figs. 1 and 2 and Table 2). The association was mildly stronger between FAQ and the tau-amyloid interaction variable than it was between FAQ and amyloid burden (pr = 0.44, *p <* 0.001) or tau deposition alone (EC tau: pr = 0.39, *p* < 0.001; IT tau: pr = 0.45, *p* < 0.001).

**Table 2:** Results of separate primary regression models (dependent variable: FAQ; independent variables: entorhinal cortex FTP SUVR, inferior temporal cortex FTP SUVR, florbetapir SUVR, EC FTP*florbetapir, IT FTP*florbetapir, age, RAVLT total learning, AMNART IQ)

		FAQ
EC tau × amyloid	Partial *R*	0.47
	*p*	< 0.001
IT tau × amyloid	Partial *R*	0.54
	*p*	< 0.001
Amyloid	Partial *R*	0.44
	*p*	< 0.001
EC tau	Partial *R*	0.39
	*p*	< 0.001
IT tau	Partial *R*	0.45
	*p*	< 0.001

### Secondary analyses

Similar results were seen when adjusting for global cognition using the MMSE as a covariate instead of RAVLT total learning (EC tau × amyloid: pr = 0.35, *p =* 0.002; IT tau × amyloid: pr = 0.38, *p* = 0.001; EC tau: pr = 0.22, *p* = 0.041; IT tau: pr = 0.22, *p =* 0.046; amyloid: pr = 0.41, *p* < 0.001) (see Table 3).

**Table 3:** Results of separate secondary regression models (dependent variable: FAQ; independent variables: entorhinal cortex FTP SUVR, inferior temporal cortex FTP SUVR, florbetapir SUVR, EC FTP*florbetapir, IT FTP*florbetapir, age, MMSE, AMNART IQ)

		FAQ
EC tau × amyloid	Partial *R*	0.35
	*p*	0.002
IT tau × amyloid	Partial *R*	0.38
	*p*	0.001
Amyloid	Partial *R*	0.41
	*p*	< 0.001
EC tau	Partial *R*	0.22
	*p*	0.041
IT tau	Partial *R*	0.22
	*p*	0.046

Similar results were seen when adding clinical status (asymptomatic CN participants vs. symptomatic combined MCI and AD dementia participants) as a covariate (EC tau × amyloid: pr = 0.44, *p <* 0.001; IT tau × amyloid: pr = 0.50, *p* < 0.001). Clinical status was also retained in the model with the symptomatic group being associated with greater IADL impairment (EC tau model: pr = 0.30, *p* = 0.008; IT tau model: pr = 0.28, *p* = 0.01) (see Table 4).

**Table 4:** Results of separate secondary regression models (dependent variable: FAQ; independent variables: entorhinal cortex FTP SUVR, inferior temporal cortex FTP SUVR, florbetapir SUVR, EC FTP*florbetapir, IT FTP*florbetapir, age, RAVLT, AMNART IQ, diagnosis)

		FAQ
EC tau × amyloid	Partial *R*	0.44
	*p*	< 0.001
IT tau × amyloid	Partial *R*	0.5
	*p*	< 0.001

**Fig. 3:**
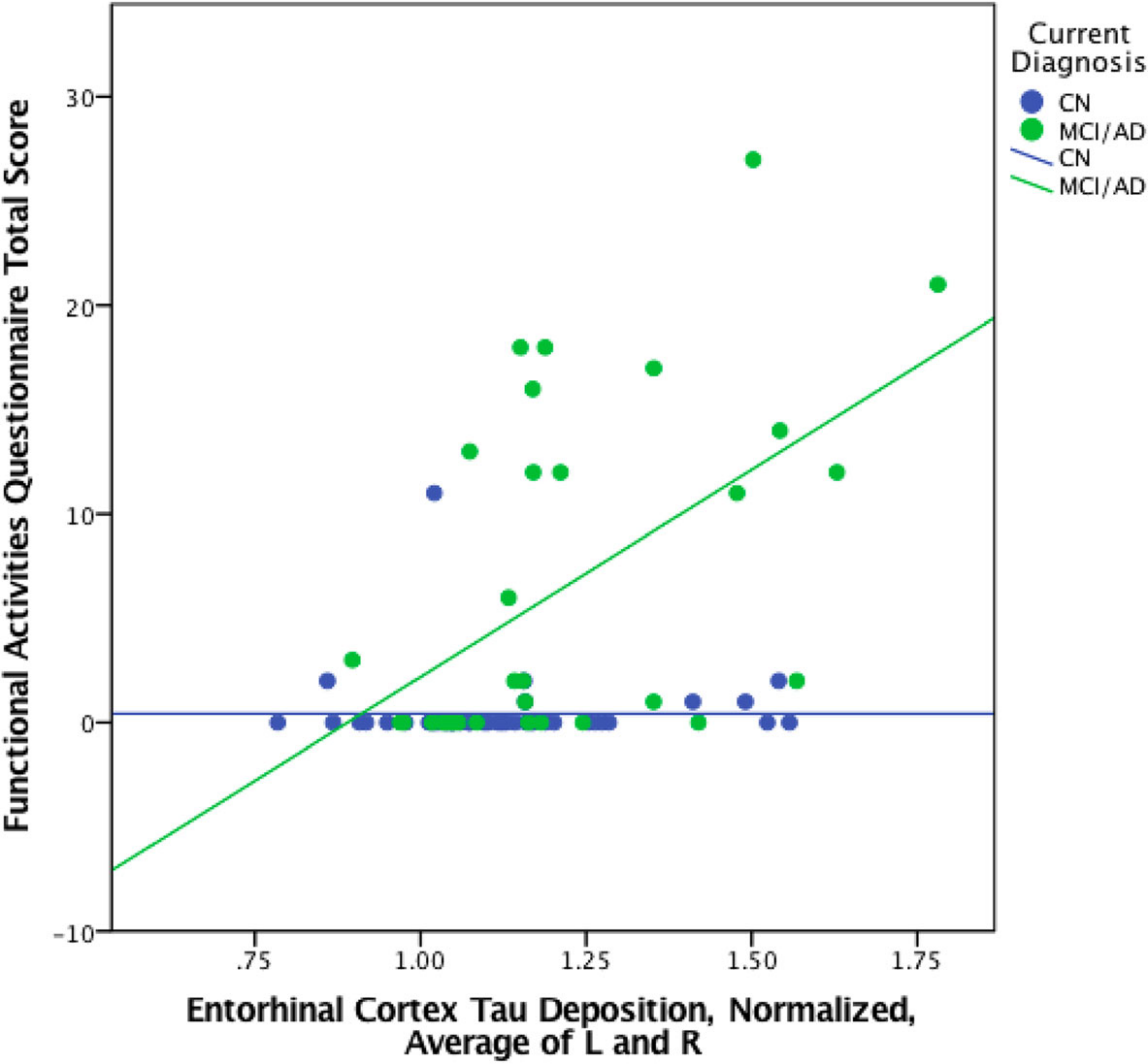
Scatter plot of entorhinal cortex FTP SUVR versus FAQ in CN, MCI, and AD dementia participants, stratified by the diagnostic group (CN, MCI/AD)

**Fig. 4:**
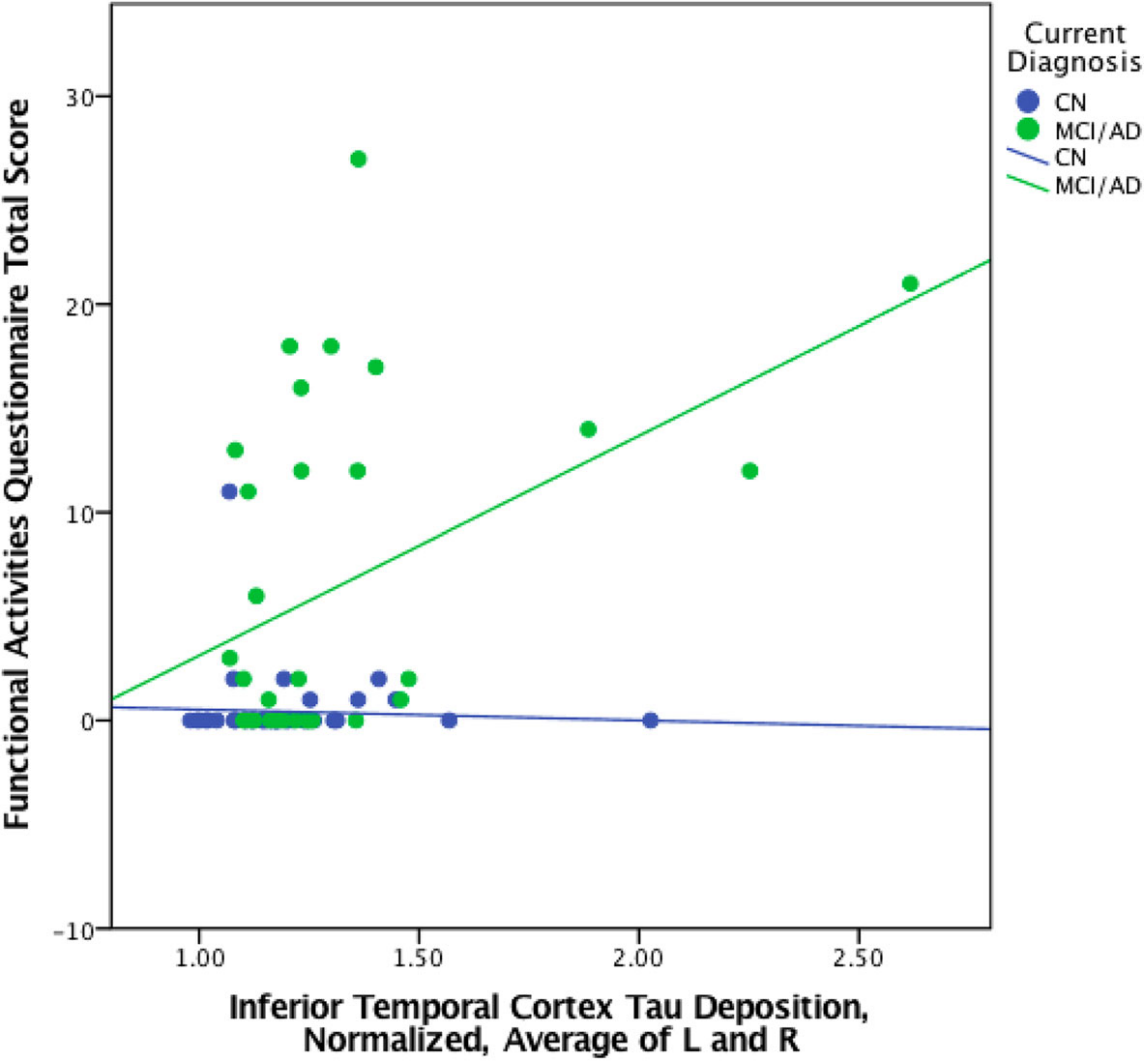
Scatter plot of inferior temporal cortex FTP SUVR versus FAQ in CN, MCI, and AD dementia participants, stratified by the diagnostic group (CN, MCI/AD)

When the same regression analyses were done with CN participants only, no significant associations were found (EC tau: pr = 0.069, *p* = 0.64; EC tau × amyloid: pr = 0.219, *p* = 0.164; IT tau: pr = 0.072, *p* = 0.629; IT tau × amyloid: pr = 0.091, *p* = 0.567) (see Figs. 3 and 4 and Table 5). However, significant associations were found among a combined group of MCI and AD dementia participants without CN participants (EC tau: pr = 0.52, *p* = 0.001; EC tau × amyloid: pr = 0.61, *p* < 0.001; IT tau: pr = 0.54, *p* = 0.001; IT tau × amyloid: pr = 0.64, *p* < 0.001) (see Figs. 3 and 4 and Table 6).

**Table 5:** Results of separate secondary regression models in CN participants (dependent variable: FAQ; independent variables: entorhinal cortex FTP SUVR, inferior temporal cortex FTP SUVR, florbetapir SUVR, EC FTP*florbetapir, IT FTP*florbetapir, age, RAVLT, AMNART IQ)

		FAQ
EC tau × amyloid	Partial *R*	0.219
	p	0.164
IT tau × amyloid	Partial *R*	0.092
	*p*	0.567
EC tau	Partial *R*	0.069
	*p*	0.64
IT tau	Partial *R*	0.072
	*p*	0.629

**Table 6:** Results of separate secondary regression models in MCI/AD dementia participants (dependent variable: FAQ; independent variables: entorhinal cortex FTP SUVR, inferior temporal cortex FTP SUVR, florbetapir SUVR, EC FTP*florbetapir, IT FTP*florbetapir, age, RAVLT, AMNART IQ)

		FAQ
EC tau × amyloid	Partial *R*	0.61
	*p*	< 0.001
IT tau × amyloid	Partial *R*	0.64
	*p*	< 0.001
EC tau	Partial *R*	0.52
	*p*	0.001
IT tau	Partial *R*	0.54
	*p*	0.001

To determine whether the association between IADL and pathologic markers is stronger among amyloid-positive participants, the same regression analyses were done in amyloid-positive participants only (*n* = 29). Significant associations were found (EC tau: pr = 0.39, *p* < 0.001; EC tau × amyloid: pr = 0.39, *p* = 0.05; IT tau: pr = 0.45, *p* < 0.001; IT tau × amyloid: *p* > 0.1) (see Table 7).

**Table 7:** Results of separate secondary regression models in amyloid-positive participants (dependent variable: FAQ; independent variables: entorhinal cortex FTP SUVR, inferior temporal cortex FTP SUVR, florbetapir SUVR, EC FTP*florbetapir, IT FTP*florbetapir, age, RAVLT, AMNART IQ)

		FAQ
EC tau × amyloid	Partial *R*	0.39
	*p*	0.05
IT tau × amyloid	Partial *R*	–
	*p*	> 0.1
EC tau	Partial *R*	0.39
	*p*	< 0.001
IT tau	Partial *R*	0.45
	*p*	< 0.001

## Discussion

The objective of this study was to understand the relationship between cortical tau and amyloid burden and IADL impairment across the AD spectrum. Results from this study suggest that greater medial and inferior temporal tau burden and cortical amyloid burden were associated with greater IADL impairment. Taken together, tau and amyloid were more strongly associated with IADL impairment than either pathologic marker alone. These findings were independent of age, cognitive reserve, and cognitive impairment.

The spatial distribution of FTP uptake found in this study in relation to FAQ resembles that seen in previous reports studying FTP in relationship to clinical symptoms across the AD spectrum. A study using data from ADNI to identify longitudinal and cross-sectional imaging correlates of FTP uptake showed that it is largely restricted to the temporal lobe in early stages of AD [17]. Johnson et al., in a study on FTP PET imaging in aging and AD, found that tau uptake in the inferior temporal lobe was associated with greater cognitive impairment [16]. A study exploring the relationship between FTP uptake and depressive symptoms in CN older adults found that greater FTP uptake in the entorhinal and inferior temporal cortex was associated with greater depressive symptoms [26].

Selection of ROIs in this study was based on the previous work outlining the relationship between IADL impairment and frontal, parietal, and inferior temporal regions [7–9, 12, 21, 27]. In this present study, a relationship between IADL and FTP uptake was only seen in the entorhinal cortex and inferior temporal cortex, which corresponds with greater tau deposition in the entorhinal cortex and inferior temporal gyrus seen in early-stage AD. Neurodegeneration in the medial temporal lobe is typically involved in the pathogenesis of AD, as is described in a recent study by Jutten et al. exploring the association between IADL impairment and the regions of cortical atrophy [27]. However, while Jutten et al. found that the parietal and frontal regions of cortical atrophy were associated with IADL impairment, we did not find associations between FTP uptake in the frontal and parietal regions and IADL impairment in our study. The use of voxel-based morphometry, though beyond the scope of the current study, may have been a more sensitive approach to identifying the relationships between IADL impairment and the frontal and parietal regions observed previously.

In our study, we included CN older adults, individuals with MCI, and individuals with AD dementia. When we adjusted our models for clinical status (asymptomatic vs. symptomatic), the association between the amyloid-tau interaction and IADL impairment remained significant. However, after the model was run separately by a diagnostic group, the association between tau and amyloid burden and IADL impairment was found in the MCI/AD dementia group but not in the CN group, suggesting that these results were driven largely by the MCI and AD dementia groups. While the predominant effect is driven by MCI and AD participants, CN participants were included in this analysis due to the small sample size of ADNI participants who underwent FTP PET imaging.

Additionally, previous studies have explored the relationship between IADL impairment and other imaging modalities across the AD spectrum, with longitudinal analyses revealing significant associations among CN participants. It was therefore important to include CN participants in this current analysis in order to evaluate cross-sectional associations between IADL impairment and FTP PET in this diagnostic group. In a study on the association between IADL impairment with 18F-fluorodeoxyglucose (FDG) PET, Roy et al. showed that the cross-sectional association between IADL impairment and FDG hypometabolism was significant in the AD dementia and MCI groups but not in the CN group; longitudinal results, however, were significant across the AD spectrum, including in CN participants [7]. Similarly, in a cross-sectional analysis of the relationship between IADL impairment and cerebrospinal fluid (CSF) amyloid-β and inferior temporal atrophy, associations were seen beginning primarily at the stage of mild dementia; longitudinally, however, these associations, as well as an association with greater CSF total tau, were significant across the AD spectrum, beginning in CN elderly at risk for AD [11]. Given the absence of longitudinal data on ADNI participants who underwent FTP PET imaging, it was not possible to run a longitudinal analysis of the relationship between IADL impairment and FTP uptake. Future longitudinal data will help clarify whether an association exists earlier in the AD spectrum.

Another notable finding in this study is that the tau-amyloid interaction is more strongly associated with IADL impairment than either amyloid or tau alone. While it is well known that there is a molecular interaction between amyloid and tau deposits in AD pathogenesis, the biochemical pathways in which they interact are yet to be fully elucidated. While several studies characterize tau as a mediator of amyloid-β-induced synaptic deficits, others have suggested that tau also plays a role in modulating amyloid-β plaque deposition [28, 29]. The results of this study support the evidence from molecular studies pointing to the interaction between tau and amyloid in AD pathogenesis, showing that the combination of amyloid and tau is a better predictor IADL impairment than either biomarker alone.

One major strength of this study is that it utilizes the results from a multi-center longitudinal cohort study with an extensive collection of clinical and imaging data. The continuation of this cohort study will allow for future longitudinal analyses exploring the relationship between IADL impairment and tau and cortical amyloid. Additionally, this study has added to the understanding of the biological underpinnings of IADL impairment with the exploration of the roles of tau and amyloid and the interaction between these neuropathological markers. That said, there are several limitations. ADNI participants were not representative of the general population since they were carefully selected to have limited health issues, psychiatric conditions, or cerebrovascular disease. They also had high premorbid intelligence and had a high proportion of apolipoprotein E ε4 carriers [2, 30]. To address this, the regression analyses carried out in this study adjusted for premorbid intelligence. Furthermore, the study population resembles that of most AD spectrum clinical trials, allowing for the comparison of these results to clinical trial outcomes. The sample size of the study participants was small due to the recent addition of FTP PET imaging in the ADNI protocol. Additionally, not enough longitudinal data have been obtained to allow for longitudinal analyses. Therefore, cross-sectional and longitudinal analyses exploring the association between IADL impairment and FTP PET must be carried out once more ADNI participants have undergone FTP PET imaging. The majority of CN participants in this study had a FAQ score of 0, creating a floor effect, with MCI and AD dementia participants driving the results in our models. As discussed earlier, however, prior studies with longitudinal analyses have shown that FAQ can detect IADL impairment early on, including in participants with a CN diagnosis at baseline who are followed over time [7, 11]. Furthermore, the FAQ itself, while useful for its convenient and quick administration in elderly populations, may not be as sensitive to subtle changes in IADL as other measures. A few subjective IADL assessments such as the Alzheimer’s Disease Cooperative Study ADL Prevention Instrument and the Cognitive Function Instrument have been shown to have questions more geared toward detecting earlier IADL changes, yielding a wider range of performance within CN participants, and avoiding the floor effect seen with the FAQ. Similarly, a few performance-based IADL tests such as the Harvard Automated Phone Task and the Czaja Functional Assessment Battery that include more complex activities and time scores have shown a wider range of IADL performance within CN participants. Finally, in the current study, the selection of ROIs was based on their correlation with IADL in previous studies, a hypothesis-driven approach. In future studies, using whole-brain voxel-based analyses could be a more sensitive, unbiased, data-driven approach to identifying associations between specific regions of FTP and florbetapir uptake and IADL impairment that were not found by using pre-analyzed ROIs. Such an approach may lead to the identification of additional regions associated with IADL impairment or confirm the results of the ROI analyses.

## Conclusions

In this cross-sectional study across the AD spectrum, greater medial and inferior temporal tau burden and cortical amyloid burden were associated with greater IADL impairment. The interaction of tau and cortical amyloid was associated more strongly with IADL impairment than either pathologic marker alone, reflecting the biological interactions of tau and amyloid in AD pathogenesis. Further longitudinal exploration of the association of tau and amyloid with IADL impairment once more data is available will allow us to better understand the biological underpinnings of IADL impairment in order to more sensitively detect functional impairment earlier in the disease.

## Abbreviations

AD: Alzheimer’s disease
ADNI: Alzheimer’s Disease Neuroimaging Initiative
AMNART: American National Adult Reading Test
CN: Clinically normal
CSF: Cerebrospinal fluid
EC: Entorhinal cortex
FAQ: Functional Activities Questionnaire
FDG: 18F-fluorodeoxyglucose
FTP: Flortaucipir
IADL: Instrumental activities of daily living
IT: Inferior temporal
LONI: Laboratory of Neuroimaging
MCI: Mild cognitive impairment
MMSE: Mini-Mental State Examination
MRI: Magnetic resonance imaging
PET: Positron emission tomography
RAVLT: Rey Auditory Verbal Learning Test
